# Early iron supplementation in exclusively breastfed Gambian infants: a randomized controlled trial

**DOI:** 10.2471/BLT.23.289942

**Published:** 2024-01-29

**Authors:** Mamadou Bah, Isabella Stelle, Hans Verhoef, Alasana Saidykhan, Sophie E Moore, Bubacarr Susso, Andrew M Prentice, Carla Cerami

**Affiliations:** a Medical Research Council Unit, The Gambia at the London School of Hygiene & Tropical Medicine, Atlantic Boulevard, Fajara, PO Box 273, Banjul, Gambia.; b Department of Women and Children's Health, King's College London, London, England.; c Division of Human Nutrition and Health, Wageningen University, Wageningen, Kingdom of the Netherlands.

## Abstract

**Objective:**

To investigate the effect of daily iron supplementation for 14 weeks on the serum iron concentration and other markers of iron status in exclusively breastfed infants in Gambia.

**Methods:**

A placebo-controlled, randomized, double-blind trial was performed in rural Gambia between 3 August 2021 and 9 March 2022. Overall, 101 healthy, exclusively breastfed infants aged 6 to 10 weeks were recruited at vaccination clinics and through community health workers. Infants were randomized to receive iron supplementation (7.5 mg/day as ferrous sulfate in sorbitol solution) or placebo for 98 days. Venous blood samples were collected at baseline and on day 99 to assess the serum iron concentration and other markers of iron and haematological status.

**Findings:**

At day 99, the serum iron concentration was significantly higher in the iron supplementation group than the placebo group (crude difference in means: 2.5 µmol/L; 95% confidence interval: 0.6 to 4.3) and there were significant improvements in other iron and haematological markers. There were 10 serious adverse events (five in each group), 106 non-serious adverse events (54 with iron supplementation; 52 with placebo) and no deaths. There was no marked difference between the groups in maternally reported episodes of diarrhoea, fever, cough, skin infection, eye infection or nasal discharge.

**Conclusion:**

In exclusively breastfed Gambian infants, iron supplementation from 6 weeks of age was associated with a significant improvement in markers of iron status at around 6 months of age. There was no indication of adverse effects on growth or infections.

## Résumé

**Objectif:**

Étudier l'effet d'une administration quotidienne de suppléments de fer pendant 14 semaines sur la concentration sérique en fer et sur d'autres marqueurs relatifs au bilan en fer chez des nourrissons exclusivement allaités au sein en Gambie.

**Méthodes:**

Un essai randomisé en double aveugle et contrôlé contre placebo a été mené en région rurale dans le pays entre le 3 août 2021 et le 9 mars 2022. Au total, 101 nourrissons sains âgés de six à dix semaines et exclusivement allaités au sein ont été recrutés dans des centres de vaccination et par le biais d'agents de santé communautaires. Les nourrissons ont reçu aléatoirement un supplément de fer (7,5 mg/jour de sulfate ferreux dans une solution de sorbitol) ou un placebo durant 98 jours. Des échantillons de sang veineux ont été récoltés au début de l'essai et au 99^e^ jour afin de mesurer la concentration sérique en fer et d'autres marqueurs relatifs au bilan en fer et au bilan hématologique.

**Résultats:**

Au 99^e^ jour, la concentration sérique en fer était considérablement plus élevée dans le groupe ayant reçu un supplément de fer que dans celui ayant reçu un placebo (différence brute moyenne: 2,5 µmol/L; intervalle de confiance de 95%: 0,6 à 4,3), tandis que d'autres marqueurs relatifs au bilan en fer et au bilan hématologique affichaient de nettes améliorations. Dix effets secondaires graves ont été signalés (cinq dans chaque groupe), 106 effets secondaires sans gravité (54 dans le groupe ayant reçu un supplément de fer; 52 dans le groupe ayant reçu un placebo) et aucun décès. Aucune différence majeure n'a été constatée entre les groupes au niveau des épisodes de diarrhée, fièvre, toux, infection cutanée, infection oculaire ou écoulement nasal signalés par la mère.

**Conclusion:**

Chez les nourrissons gambiens exclusivement allaités au sein, l'administration de suppléments de fer dès l'âge de six semaines a entraîné une nette amélioration des marqueurs relatifs au bilan en fer vers l'âge de six mois. Rien n'indique que ces suppléments aient eu des effets secondaires sur la croissance ou causé des infections.

## Resumen

**Objetivo:**

Investigar el efecto de la administración diaria de suplementos de hierro durante 14 semanas sobre la concentración sérica de hierro y otros marcadores del estado del hierro en lactantes alimentados exclusivamente con leche materna en Gambia.

**Métodos:**

Entre el 3 de agosto de 2021 y el 9 de marzo de 2022, se llevó a cabo un ensayo con enmascaramiento doble, aleatorizado y controlado con placebo en áreas rurales de Gambia. En total, se reclutaron 101 lactantes sanos, alimentados exclusivamente con leche materna y con edades comprendidas entre las 6 y las 10 semanas, en clínicas de vacunación y a través de trabajadores sanitarios comunitarios. Los lactantes se asignaron aleatoriamente para recibir suplementos de hierro (7,5 mg/día como sulfato ferroso en solución de sorbitol) o placebo durante 98 días. Se recogieron muestras de sangre venosa al inicio del ensayo y el día 99 para evaluar la concentración sérica de hierro y otros marcadores del hierro y el estado hematológico.

**Resultados:**

En el día 99, la concentración sérica de hierro era significativamente mayor en el grupo de suplementación con hierro que en el grupo placebo (diferencia bruta de medias: 2,5 µmol/L; intervalo de confianza del 95%: 0,6 a 4,3) y hubo mejoras significativas en otros marcadores hematológicos y de hierro. Ocurrieron 10 acontecimientos adversos graves (cinco en cada grupo), 106 acontecimientos adversos no graves (54 con suplementos de hierro; 52 con placebo) y ninguna muerte. No hubo diferencias notables entre los grupos respecto a los episodios de diarrea, fiebre, tos, infección cutánea, infección ocular o secreción nasal notificados por las madres.

**Conclusión:**

En los lactantes de Gambia alimentados exclusivamente con leche materna, la administración de suplementos de hierro a partir de las 6 semanas de vida se asoció a una mejora significativa de los marcadores del estado del hierro en torno a los 6 meses de edad. No hubo indicios de efectos adversos sobre el crecimiento o las infecciones.

## ملخص

**الغرض:**

دراسة تأثير مكملات الحديد الغذائية اليومية لمدة 14 أسبوعًا على تركيز الحديد المصلي، والعلامات الأخرى لحالة الحديد عند الرضع المعتمدين على الرضاعة الطبيعية بشكل حصري في غامبيا.

**الطريقة:**

تم إجراء تجربة عشوائية، مزدوجة التعمية، بمساعدة علاج وهمي في الريف الغامبي في الفترة ما بين 3 أغسطس/آب 2021، و9 مارس/آذار 2022. تم الاستعانة بشكل عام بعدد 101 رضيعًا، يتمتعون بصحة جيدة، ويتلقون رضاعة طبيعية بشكل حصرية، تتراوح أعمارهم من 6 إلى 10 أسابيع، في عيادات التطعيم ومن خلال العاملين في مجال الصحة المجتمعية. تم اختيار الرضع بشكل عشوائي لتلقي مكملات الحديد الغذائية (7.5 ملغ/يوم على شكل كبريتات الحديدوز في محلول السوربيتول)، أو الدواء الوهمي لمدة 98 يومًا. تم جمع عينات الدم من الوريد عند خط الأساس وفي اليوم 99، لتقييم تركيز الحديد المصلي، وغيره من علامات حالة الحديد والدم.

**النتائج:**

في اليوم 99، كان تركيز الحديد المصلي أعلى بشكل ملموس في مجموعة مكملات الحديد الغذائية، عنه في مجموعة الدواء الوهمي (الفارق العام في المتوسطات: 2.5 ميكرومول/لتر؛ بفاصل ثقة مقداره %95: 0.6 إلى 4.3) وكانت هناك تحسينات ملموسة في الحديد وعلامات الحديد الأخرى في الدم. كانت هناك 10 آثار جانبية خطيرة (خمسة في كل مجموعة)، و106 أثرًا جانبيا غير خطير (54 مع مكملات الحديد الغذائية، 52 مع الدواء الوهمي) ولم تحدث وفيات. ولم يكن هناك فرق ملحوظ بين المجموعتين في نوبات الإسهال، أو الحمى، أو السعال، أو عدوى الجلد، أو عدوى العين، أو إفرازات الأنف، التي تم الإبلاغ عنها.

**الاستنتاج:**

بالنسبة للرضع الغامبيين الذين يتلقون الرضاعة الطبيعية بشكل حصري، ارتبطت مكملات الحديد الغذائية اعتباراً من عمر 6 أسابيع بتحسن كبير في علامات حالة الحديد عند حوالي 6 أشهر من العمر. ولم يكن هناك مؤشر على وجود آثار سلبية على النمو أو حدوث العدوى.

## 摘要

**目的:**

探讨在冈比亚纯母乳喂养的婴儿中，连续 14 周每日补铁对其血清铁浓度和其他铁状态指标的影响。

**方法:**

在 2021 年 8 月 3 日至 2022 年 3 月 9 日期间，我们在冈比亚农村地区开展了一项设有安慰剂对照的随机双盲试验。我们通过疫苗接种诊所以及社区卫生工作者共招募了 101 名 6 至 10 周龄的纯母乳喂养的健康婴儿。婴儿随机接受铁补充剂（含有硫酸亚铁的山梨醇溶液，每天服用 7.5 毫克）或接受安慰剂，持续时间为 98 天。在基线和第 99 天采集静脉血样本，对血清铁浓度以及其他铁和血液状态指标进行评估。

**结果:**

在第 99 天，补铁组婴儿的血清铁浓度显著高于安慰剂组（粗差均值：2.5 微摩尔/升；95% 置信区间：0.6 至 4.3），并且其他铁和血液指标也有显著改善。有严重不良事件 10 例（每组 5 例），非严重不良事件 106 例（补铁组 54 例；安慰剂组 52 例），无死亡事件。关于母亲报告腹泻、发烧、咳嗽、皮肤感染、眼部感染或流鼻涕的情况，两组之间没有显著差异。

**结论:**

在进行纯母乳喂养的冈比亚婴儿中，从 6 周龄开始补铁与其在 6 个月龄左右时铁状态指标的显著改善有很大关系。没有迹象表明会对生长或感染产生不良影响。

## Резюме

**Цель:**

Изучить влияние ежедневного приема добавок железа в течение 14 недель на концентрацию железа в сыворотке крови и другие маркеры содержания железа в организме гамбийских младенцев, находящихся исключительно на грудном вскармливании.

**Методы:**

Двойное слепое рандомизированное плацебо-контролируемое исследование проводилось в сельской местности Гамбии в период с 3 августа 2021 года по 9 марта 2022 года. В общей сложности в центрах вакцинации и через медико-санитарных работников был включен 101 здоровый, находящийся исключительно на грудном вскармливании младенец в возрасте от 6 до 10 недель. Младенцы были рандомизированы для получения добавок железа (7,5 мг/день в виде сульфата железа в растворе сорбита) или плацебо в течение 98 дней. Образцы венозной крови собирали на исходном уровне и на 99-й день для оценки концентрации железа в сыворотке и других маркеров железа и гематологического статуса.

**Результаты:**

На 99-й день концентрация железа в сыворотке крови была значительно выше в группе, принимавшей железо, по сравнению с группой плацебо (приблизительная разница в средних значениях: 2,5 мкмоль/л; 95%-й ДИ: от 0,6 до 4,3), а также наблюдалось значительное улучшение других маркеров железа и гематологических показателей. Было зарегистрировано 10 серьезных нежелательных явлений (по пять в каждой группе), 106 несерьезных нежелательных явлений (54 при приеме добавок железа; 52 при приеме плацебо), и не было зарегистрировано ни одного летального исхода. Между группами не было выявлено заметной разницы в количестве зарегистрированных матерью эпизодов диареи, лихорадки, кашля, кожных инфекций, глазных инфекций или выделений из носа.

**Вывод:**

У гамбийских младенцев, находящихся исключительно на грудном вскармливании, прием добавок железа с 6-недельного возраста сопровождался значительным улучшением маркеров содержания железа в организме в возрасте около 6 месяцев. Не было выявлено негативного влияния на рост или развития инфекций.

## Introduction

Breastfeeding infants offers strong protection against infection and associated deaths, but there are increasing doubts about whether the iron supplied by exclusive breastfeeding in low-income countries is adequate for the infant’s requirements during the first 6 months of life.^1^^,^^2^ As the iron content of human milk is only around 0.35 mg/L,^3^ an infant typically receiving 750 to 1000 mL of breastmilk per day will have a daily iron intake of 0.25 to 0.35 mg. The Institute of Medicine in the United States of America assumed that breastmilk iron is adequate and set a requirement of 0.27 mg/day for the first 6 months of life.^4^ After 6 months, the recommendation jumps around 40-fold to 11 mg/day because of iron loss and needs for growth.^4^ However, this abrupt jump is not physiological and back-extrapolation to infants younger than 6 months, when they are growing fast, suggests a true iron dietary requirement of at least 10 mg/day. Although breastmilk iron is largely bound to lactoferrin and is more readily absorbed than non-milk iron, the quantity provided is an ten times below that required.^3^ Exclusively breastfed infants, therefore, must draw on their endowed iron reserves (i.e. liver ferritin and recycled iron from fetal haemoglobin) for their daily needs, for growth and, especially, for their expanding red cell mass.^2^

The fetus accrues most of its iron during the final trimester of pregnancy and is, therefore, dependent on the mother’s iron status.^5^ Infants born prematurely, with a low birth weight or to iron-deficient mothers, and those whose umbilical cord is clamped soon after birth, start life with smaller iron stores. They may therefore be less able to meet iron requirements for growth and metabolism while being breastfed. In particular, neurones and cell lineages involved in adaptive immunity have a high requirement for iron, which can be accessed only from circulating plasma.^6^ Consequently, some paediatricians in many high-income countries recommend supplemental iron (2 to 4 mg/kg orally) for breastfed infants, starting soon after birth.^3^

When recommending that infants be exclusively breastfed until 6 months of age, a World Health Organization (WHO) expert panel acknowledged that many children could become iron-deficient without supplemental iron, but offered no solution.^7^ Infants in low-income countries are more likely to be born with low iron reserves and to be exclusively breastfed.^8^ For example, a previous study of breastfed Gambian children found that at 2 months of age functional indicators of iron deficiency (i.e. transferrin and soluble transferrin receptor concentrations) started to rise, haemoglobin levels fell and the proportion of anaemic children increased.^2^ Moreover, the mean serum iron concentration, which was within the standard range at birth (i.e. around 15 μmol/L), fell rapidly to below 8 μmol/L at 2 months, and to 2 to 4 μmol/L by 6 months – both well below reference levels for well-nourished children (i.e. 10 to 20 μmol/L).^2^

The aim of our proof-of-principle, placebo-controlled, randomized trial was to determine whether the daily administration of 7.5 mg of iron as ferrous sulfate for 14 weeks to exclusively breastfed Gambian infants aged 6 to 10 weeks improves their iron status. We monitored the serum iron concentration, other iron and haematological markers and adverse events.

## Methods

We conducted a double-blind, randomized, placebo-controlled trial in rural Jarra West district in the Lower River Region of Gambia from 3 August 2021 to 9 March 2022. Currently, malaria is almost non-existent in this area: the 2019 Demographic and Health Survey reported that only 0.4% of children younger than 5 years had a *Plasmodium* infection.^9^ Full details of the study design are available in the published protocol.^10^

Infants aged 6 to 10 weeks were identified at vaccination clinics and in the community by fieldworkers and local health workers. All participants were generally healthy and were exclusively breastfed as reported by their mothers. In the study, biological sex was determined by mothers’ reports. No attempt was made to assess gender. After obtaining written informed consent, participants were invited to the Jarra Soma Health Centre and screened by research nurses for eligibility, which included a willingness to stay in the participating community and to adhere to the trial protocol. Formula-fed infants and those with fever, a haemoglobin level < 7.0 g/dL, or acute or chronic illness were excluded. All infants were provided with mosquito nets to decrease the risk of malaria.

At baseline (day 0), each infant’s weight was measured to the nearest 10 g using a Seca 336 infant scale (Seca, Hamburg, Germany) and each infant’s length was measured to the nearest 1 mm using a Seca 417 measuring board (Seca, Hamburg, Germany). Eligible children were randomized on day 0 or 1 (after haemoglobin measurement) to either iron supplementation or placebo. Permuted block randomization, with a fixed block size of six and an allocation ratio of 1:1 was used. Randomization codes were produced using a random number generator in RStudio v. 4.2.1 (Posit, Boston, United States of America) and the codes were uploaded into a REDCap database (Vanderbilt University, Nashville, USA). Iron and placebo groups were subdivided into three subgroups each to further conceal the treatment allocation, and the randomization code was used to assign each participant to one of the six subgroups. The randomization code was unblinded only after data were cleaned and locked.

Infants received either a ferrous sulfate supplement (7.5 mg/day of iron) in 0.5 mL of 70% sorbitol solution (Pharmacy Innovations, Jamestown, USA) or 0.5 mL of 70% sorbitol solution only. Solutions were repackaged into dark identical bottles to ensure blinding. Supplementation was given by fieldworkers from day 1 to day 98 (14 weeks) with the support of parents and guardians. The storage temperature of the solutions was monitored. Daily questionnaires on iron supplementation and health, and weekly questionnaires on infant feeding were completed by fieldworkers throughout the 98 days. In addition, post-supplementation health status was assessed daily up to day 112 (i.e. 16 weeks).

### Outcomes

The primary study endpoint was the infant’s serum iron concentration at day 99, which allowed for one day of wash-out. Secondary endpoints included anaemia, iron deficiency, iron deficiency anaemia and concentrations of erythroferrone, erythropoietin, hepcidin and other iron and haematological markers at day 99 (full list in Table 1).

**Table 1:** Baseline characteristics of participants, study of early iron supplementation in breastfed infants, Gambia, 2021–2022

Baseline characteristic^a^	Iron supplementation group (*n* = 50)	Placebo group (*n* = 51)
**Infants in age range, no. (%)**		
6 to < 7 weeks	29 (58.0)	25 (49.0)
7 to < 8 weeks	12 (24.0)	12 (23.5)
8 to < 9 weeks	3 (6.0)	8 (15.7)
≥ 9 weeks	6 (12.0)	6 (11.8)
**Female sex, no. (%)**	26 (52.0)	29 (56.9)
**No. children born to mother, mean (SD)**	3.9 (2.2)	3.9 (2.1)
**Serum iron in µmol/L, mean (SD)**	12.6 (4.5)	12.5 (6.9)
**Serum ferritin in µg/L, geometric mean (GSD)**	283.2 (1.81)	262.6 (1.76)
**Serum soluble transferrin receptor in mg/L, geometric mean (GSD)**	2.81 (1.28)	2.76 (1.30)
**Serum soluble transferrin receptor /log_10_ ferritin index, geometric mean (GSD)**	1.15 (1.34)	1.15 (1.31)
**Serum transferrin in g/L, mean (SD)**	2.2 (0.4)	2.1 (0.4)
**Unsaturated iron binding capacity in µmol/L, mean (SD)**	37.0 (11.0)	35.2 (11.0)
**Percentage transferrin saturation, geometric mean (GSD)**	24.0 (1.65)	22.6 (2.00)
**Haemoglobin in g/dL, mean (SD)**	10.9 (1.6)	11.0 (1.2)
**Mean corpuscular volume in fL, mean (SD)**	89.6 (5.3)	89.2 (5.4)
**Mean corpuscular haemoglobin in pg/cell, mean (SD)**	29.4 (1.7)	28.9 (2.1)
**Mean corpuscular haemoglobin concentration in g/dL, mean (SD)**	32.9 (1.4)	32.6 (1.3)
**Reticulocyte count in cells/L × 10**^10^ **geometric mean (GSD)**	0.07 (1.44)	0.07 (1.49)
**Reticulocyte count as % of red blood cells,^b^ geometric mean (GSD)**	1.83 (1.57)	1.91 (1.54)
**Immature reticulocyte fraction in %,^b^ geometric mean (GSD)**	17.2 (1.50)	16.4 (1.52)
**Reticulocyte haemoglobin in pg/cell,^b^ mean (SD)**	31.8 (1.8)	31.3 (2.4)
**Serum hepcidin in µg/L, geometric mean (GSD)**	20.3 (2.21)	19.6 (2.36)
**Serum erythropoietin in IU/L, geometric mean (GSD)**	12.7 (2.05)	10.8 (1.85)
**Serum α_1_-acid glycoprotein in g/L, geometric mean (GSD)**	0.66 (1.48)	0.61 (1.80)
**Infants with specified serum erythroferrone level,^c^ no. (%) **		
≤ 0.16 µg/L (limit of quantification)	33 (66.0)	38 (74.5)
> 0.16 to < 0.5 µg/L	11 (22.0)	2 (3.9)
0.5 to < 1.0 µg/L	5 (10.0)	3 (5.9)
≥ 1.0 µg/L	1 (2.0)	8 (15.7)
**Infants with specified serum CRP level, no. (%)**		
≤ 0.60 mg/L (limit of quantification)	34 (68.0)	33 (64.7)
> 0.60 to < 5 mg/L	12 (24.0)	12 (23.5)
5 to < 15 mg/L	2 (4.0)	1 (2.0)
≥ 15 mg/L	2 (4.0)	5 (9.8)
**Height-for-age *z*-score, mean (SD)**	−0.59 (1.18)	−0.68 (1.59)
**Weight-for-age *z*-score, mean (SD)**	−0.71 (1.19)	−0.70 (1.25)
**Weight-for-height *z*-score, mean (SD)**	−0.22 (1.53)	−0.11 (1.11)

CRP: C-reactive protein; GSD: geometric standard deviation; SD: standard deviation; IU: international unit.

^a^ The geometric standard deviation is a dimensionless, multiplicative factor; dividing or multiplying the geometric mean by this factor produces a variation equivalent to the subtraction or addition, respectively, of one standard deviation on a log-transformed scale.

^b^ Reticulocyte number, reticulocyte percentage, the immature reticulocyte fraction and reticulocyte haemoglobin content were not measured for 17 children (nine in the iron supplementation group and eight in the control group) due to delays in equipment installation and calibration.

^c^ Excluding one observation with an outlying value of 86.8.

At baseline and on day 99, venous blood samples (3.0 mL) were collected and stored on ice until analysis. The full blood count and reticulocyte parameters were assessed within 4 hours of collection using a Sysmex XN-1500 automated haematology analyser (Sysmex Corporation, Kobe, Japan), and a serum sample was stored at –80 °C for the subsequent measurement of iron and inflammation markers. Iron, ferritin, soluble transferrin receptor, C-reactive protein and α_1_-acid glycoprotein concentrations, unsaturated iron binding capacity and transferrin saturation were assessed using a Cobas Integra 400 plus automated biochemistry analyser (Roche Diagnostics, Rotkreuz, Switzerland). In addition, the following parameters were all analysed by enzyme-linked immunosorbent assay (ELISA): serum hepcidin using a DRG Hepcidin 25 (bioactive) HS ELISA (EIA-5782) test (DRG instruments GmbH, Marburg, Germany); erythropoietin using a Human Erythropoietin ELISA Kit ab274397 (Abcam, Cambridge, United Kingdom of Great Britain and Northern Ireland); and erythroferrone using an Intrinsic Erythroferrone IE test (Intrinsic Life Sciences, La Jolla, USA).

Safety was monitored during the intervention period by daily assessment of morbidity and adverse events. An adverse event was defined as any untoward or unfavourable medical occurrence, whether considered related to the child’s participation in the study or not. Serious adverse events were defined as adverse events that were life-threatening, resulted in death, required hospitalization or the prolongation of hospitalization, or resulted in a persistent and significant disability or incapacity – these events were always investigated by the trial clinician. In addition, we asked mothers daily about the occurrence of specific events of interest, such as diarrhoea, fever, cough, vomiting, skin infection, eye infection and nasal discharge.

### Statistical analysis

Sample size was calculated by assuming a geometric standard deviation (SD; a dimensionless variable) for the serum iron concentration of 2.39 for both iron supplementation and placebo groups, based on the results of a previous study.^2^ Assuming a drop-out rate less than 10%, a recruitment target of 100 infants in total would ensure with 80% probability that an increase in the geometric mean serum iron concentration of at least 70% in the intervention group relative to the placebo group would exclude no effect (i.e. a ratio of geometric means of 1) from the 95% confidence interval (CI). Assuming a geometric mean serum iron concentration in the placebo group of 4.3 μmol/L,^2^ an increase of 70% corresponds to +3.0 µmol/L.

#### Definitions

Transferrin saturation was calculated as the serum iron concentration in µmol/L divided by the total iron-binding capacity in µmol/L ×  100. Anaemia was defined as a haemoglobin concentration below 11.0 g/dL, even though there is no agreed definition for infants aged under 6 months.^11^ Anthropometric *z*-scores were derived using WHO’s international growth reference.^12^ Adherence was defined as the percentage of the 98 scheduled days that the supplement or placebo was observed to be ingested.

#### Intervention effects

We estimated the crude effects of the intervention on continuous outcomes using simple linear regression. We used multiple linear regression models to estimate intervention effects adjusted for the value of the specific outcome variable at baseline, sex (binary variable), age (continuous variable) and baseline haemoglobin, serum ferritin and serum soluble transferrin receptor concentrations (all continuous variables). For anthropometric outcomes, adjustment was made for the value of the outcome variable at baseline, sex and age alone. In these analyses, continuous variables were entered as fractional polynomials to allow for possible nonlinear relationships with the outcome.^13^ In all models, we accounted for heteroscedasticity using robust variance estimators. Intervention effects are reported as absolute differences in the means of normally distributed outcomes or as relative differences in the geometric means of log-transformed outcomes. If log-transformation failed to normalize the distribution of residuals within groups, we assessed group differences using the two-sample Wilcoxon rank-sum test (i.e. the Mann–Whitney *U* test). In per protocol analyses, we used logistic regression models with the *adjrr* command in Stata package *st0306.pkg* (StataCorp LLC, College Station, USA) to estimate prevalence differences adjusted for baseline characteristics.

The primary analysis was a modified intention-to-treat analysis; that is, randomized children withdrawn before their first supplemental iron or placebo dose were excluded. As such withdrawals did not occur in practice, the primary analysis was de facto by intention to treat. To account for missing values, we employed multiple imputation. In our multiple imputation model, we used all variables in the data set except for censored variables (i.e. serum erythroferrone and C-reactive protein concentrations, both of which were measured at baseline and at the end of the survey), and passive variables derived from original variables (e.g. body mass index, which was calculated from height and length; and transferrin saturation, which was calculated from the serum iron and unsaturated iron binding capacity concentrations). We performed 100 imputations for missing values in multiple variables iteratively by using chained equations. This method was done separately within intervention groups using predictive mean matching based on the nearest three neighbouring values. As supportive analyses, we also report per protocol analyses with pairwise deletion of missing values.

#### Adverse events

For adverse events and specific events of interest, we conducted a per protocol analysis. Adverse events were reported as the number of events in each treatment group. However, no formal statistical analysis was performed because the number of adverse events was small. For daily specific events of interest, we defined a new episode as one or more successive days of reported symptoms that were separated from a previous episode by 5 days without symptoms of diarrhoea, and by 3 symptom-free days for fever, cough, vomiting, skin infection, eye infection and nasal discharge. Thus, individual children could have recurrent episodes. Details of case definitions and the analysis are provided in the online repository.^14^^,^^15^

Analyses were conducted using Stata v. 17 and R v. 4.2.1 (The R Foundation, Vienna, Austria). We visually inspected histograms of variables and residuals for each group to assess the shape of their distribution and to identify possible outliers. Normally distributed variables were described using the mean and SD. Skewed variables were normalized by log transformation and described using the geometric mean and geometric SD (calculated as the exponentiated SD of the log-transformed variable). Categorical variables were described using proportions or percentages.

#### Ethics

The study was approved by the Scientific Coordinating Committee of the Medical Research Council (MRC) Unit The Gambia, the Joint Gambia Government MRC Ethics Committee, and the Ethics Committee at the London School of Hygiene and Tropical Medicine (SCC19092). The trial was conducted according to the principles of good clinical practice, had oversight from a data safety and monitoring board and a trial steering committee, and was monitored by the clinical trials office of the MRC Unit The Gambia. No interim efficacy analysis was done. The trial was registered with ClinicalTrials.gov (NCT04751994).

## Results

**Fig. 1:**
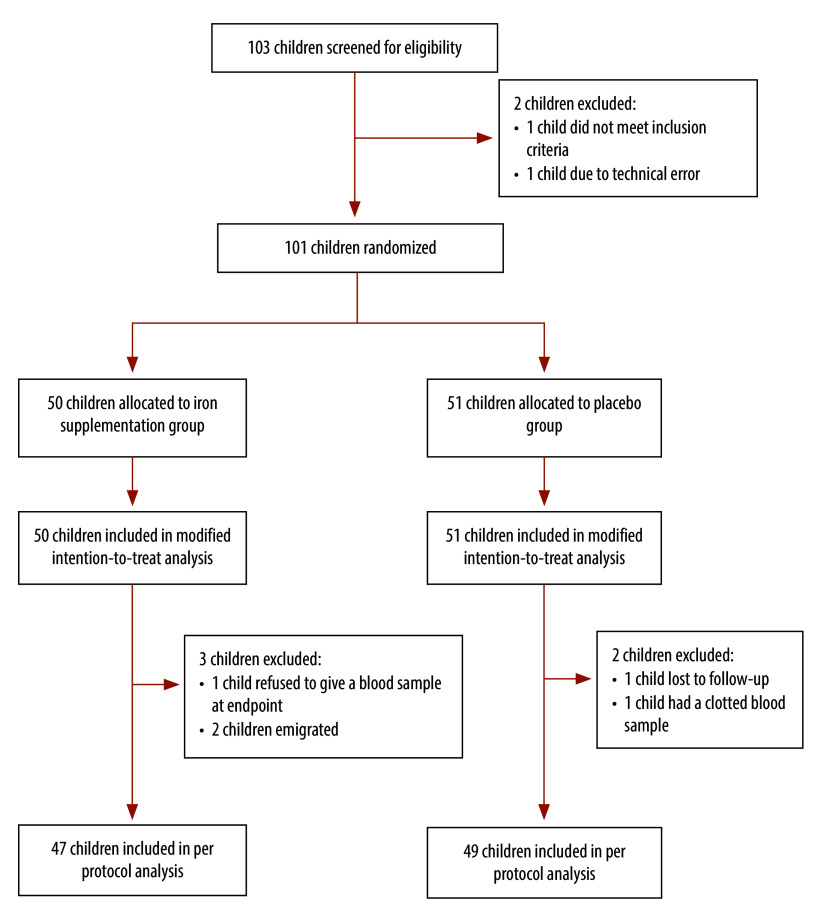
Flowchart, study of early iron supplementation in breastfed infants, Gambia, 2021–2022

In total, 103 children were invited to take part in the study, of whom 101 were enrolled: 50 (26 female) were randomized to iron supplementation and 51 (29 female) received placebo. Five participants were excluded from the final analysis (Fig. 1). At baseline, reticulocyte number, reticulocyte percentage, the immature reticulocyte fraction and reticulocyte haemoglobin content were not measured for 17 children (nine in the iron supplementation group and eight in the control group) due to delays in equipment installation and calibration. Baseline characteristics were similar in the two groups (Table 1). Overall, the adherence rate was 91.0%: it was similar in the iron supplementation and placebo groups, at 92.3 and 89.1%, respectively. The majority of non-adherence events were due to participants travelling outside the study area (details available from the online repository).^14^

The results of the intention-to-treat analysis are shown in Table 2 and the results of the per protocol analysis are available from the online repository.^14^ By day 99, the mean serum iron concentration was higher in the iron supplementation group than the placebo group (crude difference in means: 2.5 µmol/L; 95% CI: 0.6 to 4.3). After adjustment for baseline variables, the results were similar (Table 2), which indicates there was no confounding due to differences in baseline variables. All other markers of iron status were also better in the treatment group than the control group (Table 2), including: (i) a lower soluble transferrin receptor concentration (crude difference in geometric means: −7.2%; 95% CI: −10.5 to −3.7); (ii) a lower unsaturated iron binding capacity (crude difference in means: −8.7 µmol/L; 95% CI: −13.1 to −4.3); (iii) a higher transferrin saturation (crude difference in geometric means: 45.4%; 95% CI: 18.8 to 78.0); (iv) a higher ferritin concentration (crude difference in geometric means: 111.2%; 95% CI: 49.4 to 198.6); and (v) a higher hepcidin concentration (crude difference in geometric means: 79.0%; 95% CI: 20.4 to 165.9). In addition, there was a strong indication that the erythroferrone concentration was higher in the placebo group (*P*-value: 0.005).

**Table 2:** Outcomes after 98 days of iron supplementation, intention-to-treat analysis, study of early iron supplementation in breastfed infants, Gambia, 2021–2022

Outcome variable	Mean (SD) on day 99^a,b^		Value (95% CI)	
	Iron supplementation (*n* = 50)	Placebo (*n* = 51)	Unadjusted difference between groups	Adjusted difference between groups^c^
Serum iron, µmol/L	11.8 (4.9)	9.4 (4.4)	2.5 (0.6 to 4.3)	2.3 (0.5 to 4.1)
Serum ferritin, µg/L	94.0 (1.98)^d^	44.5 (2.83)^d^	111.2% (49.4 to 198.6)^e^	115.8% (60.9 to 189.4)^e^
Serum soluble transferrin receptor, mg/L	2.75 (1.11)^d^	2.96 (1.09)^d^	−7.2% (−3.7 to −10.5)^e^	−7.7% (−4.6 to −12.1)^e^
Serum soluble transferrin receptor/log_10_ ferritin index	1.41 (1.21)^d^	1.79 (1.27)^d^	−21.3% (−27.7 to −14.3)^e^	−22.7% (−28.6 to −16.4)^e^
Serum transferrin, g/L	2.39 (0.40)	2.72 (0.51)	−0.33 (−0.51 to −0.15)	0.37 (0.21 to 0.53)
Unsaturated iron binding capacity, µmol/L	47.1 (10.4)	55.8 (12.0)	−8.7 (−13.0 to −4.3)	−9.5 (−13.5 to −5.5)
Transferrin saturation, %	18.6 (1.59)^d^	12.8 (1.74)^d^	5.8 (2.7 to 8.5)	5.72 (2.84 to 8.59)
Haemoglobin, g/dL	11.2 (1.2)	10.6 (0.9)	0.6 (0.2 to 1.0)	0.6 (0.2 to 1.0)
Anaemia (i.e. haemoglobin < 11.0 g/dL), %^f^	42.7	66.9	−24.2 (−43.7 to −4.7)^e^	ND^g^
Mean corpuscular volume, fL	77.7 (4.0)	74.9 (4.4)	2.8 (1.1 to 4.5)	2.7 (1.4 to 4.1)
Mean corpuscular haemoglobin, pg/cell	24.5 (1.4)	23.3 (1.5)	1.2 (0.6 to 1.8)	1.2 (0.6 to 1.7)
Mean corpuscular haemoglobin concentration, g/dL	31.6 (0.8)	31.2 (0.9)	0.40 (0.10 to 0.80)	0.34 (0.03 to 0.64)
Reticulocyte count, cells/L × 10^9^	0.06 (1.46)^d^	0.05 (1.44)^d^	19.8 (3.4 to 38.8)^e^	23.5 (6.4 to 43.3)^e^
Reticulocyte count as % of red blood cells	1.36 (1.48)^d^	1.14 (1.45)^d^	19.4 (2.7 to 38.9)^e^	23.2 (5.5 to 43.8)^e^
Immature reticulocyte fraction, %	9.6 (1.68)^d^	10.4 (1.48)^d^	−7.9 (−23.3 to 10.5)^e^	−8.5 (−24.1 to 10.3)^e^
Reticulocyte haemoglobin, pg/cell	30.1 (2.7)	27.4 (3.1)	2.8 (1.6 to 3.9)	2.72 (1.55 to 3.90)
Serum hepcidin, µg/L	13.5 (2.19)^d^	7.6 (3.26)^d^	79.0 (20.4 to 165.9)^e^	72.4 (14.5 to 159.6)^e^
Serum erythropoietin, IU/L	8.66 (2.17)^d^	10.59 (1.73)^d^	−18.2 (−37.3 to 6.4)^e^	−18.7 (−37.3 to 5.3)^e^
Serum α_1_-acid glycoprotein, g/L	0.92 (1.52)^d^	1.01 (1.52)^d^	−9.8 (−23.5 to 6.4)^e^	−8.8 (−22.7 to 7.5)^e^
Serum erythroferrone,^h^ µg/L	0.16 (0.16 to 0.58)^i^	0.42 (0.16 to 1.26)^h^	0.005^j^	ND
Serum CRP,^k^ mg/L	1.3 (0.7 to 4.2)^i^	1.4 (0.6 to 4.6)^h^	0.93^j^	ND
Height-for-age *z*-score	−0.81 (1.24)	−0.68 (1.18)	−0.14 (−0.62 to 0.35)	−0.21 (−0.16 to 0.57)
Weight-for-age *z*-score	−0.64 (1.27)	−0.70 (1.13)	0.05 (−0.42 to 0.53)	0.02 (−0.37 to 0.41)
Height-for-weight *z*-score	−0.07 (1.11)	−0.24 (1.10)	0.18 (−0.26 to 0.61)	0.19 (−0.23 to 0.60)

CI: confidence interval; CRP: C-reactive protein; NA: not applicable; ND: not determined; SD: standard deviation; IU: international units.

^a^ All values are mean and standard deviations, except where otherwise indicated.

^b^ The analysis involved multiple imputation for missing values.

^c^ Adjustment was made for the baseline value of the outcome variable, sex, age, baseline haemoglobin concentration and baseline serum ferritin and soluble transferrin receptor concentrations; for anthropometric outcomes, adjustment was made for the baseline value of the outcome variable, sex and age only.

^d^ Geometric mean and standard deviation.

^e^ The value is percentage.

^f^ Percentage of infants.

^g^ No estimate is reported because, to our knowledge, there is no available method for computing differences in proportions adjusted for covariates with multiple imputed data.

^h^ Serum erythroferrone was left-censored at the limit of quantification (i.e. 0.16 µg/L); as 26 of 47 values in the iron supplementation group were < 0.16 µg/L, the median equals the 25th percentile.

^i^ Median and interquartile range.

^j^ The Wilcoxon rank-sum test does not give a median difference, instead *P*-value is shown.

^k^ Serum C-reactive protein was left-censored at the limit of quantification (i.e. 0.60 µg/L).

**Fig. 2:**
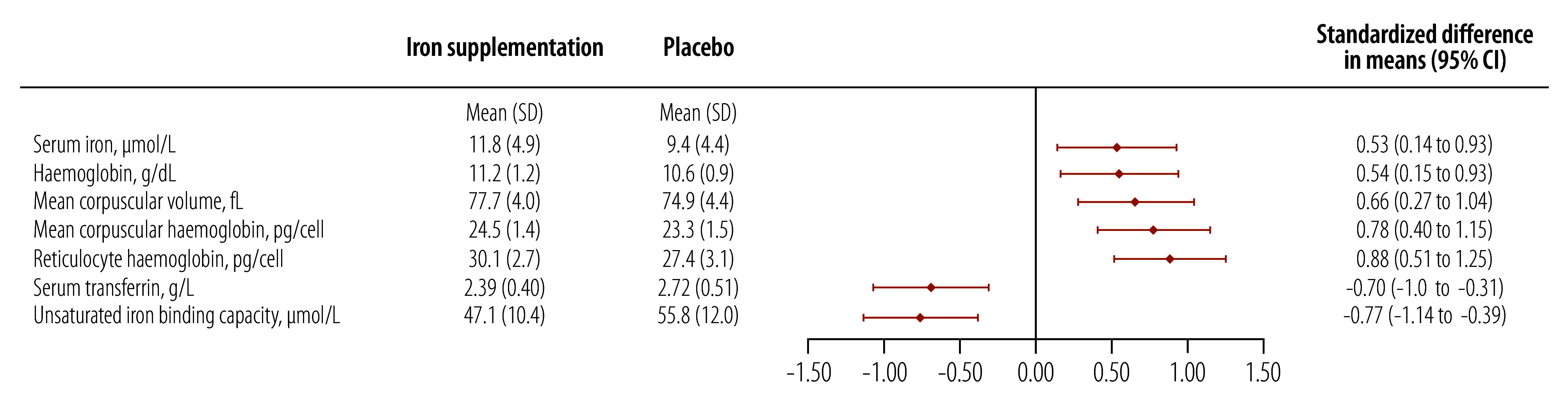
Difference between treatment groups in means of selected outcomes on day 99, study of early iron supplementation in breastfed infants, Gambia, 2021–2022

CI: confidence interval; SD: standard deviation.

Notes: For outcomes with normally distributed residuals, effect sizes were standardized (i.e. they were computed as the difference in group means divided by the standard deviation). Reductions in transferrin and unsaturated iron-binding capacity concentrations indicate improved iron status.

**Fig. 3:**
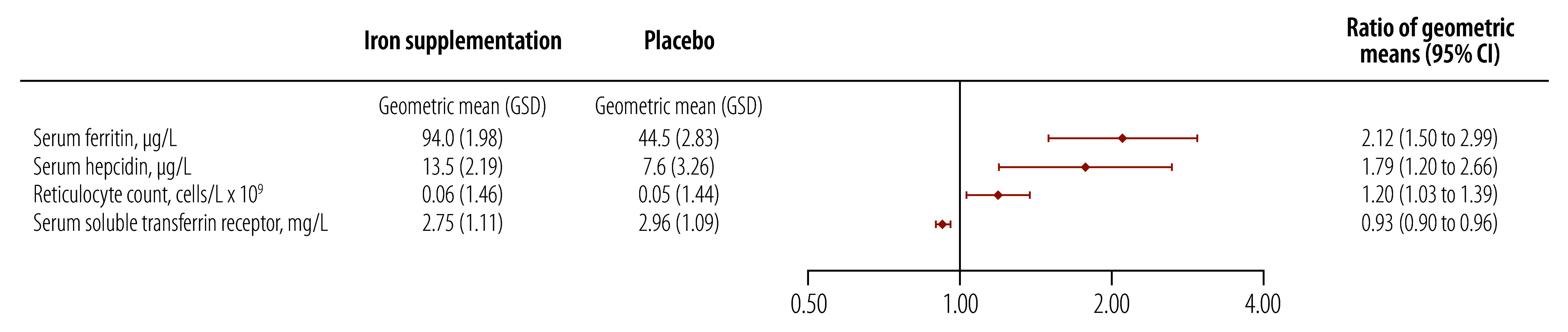
Difference between treatment groups in geometric means of selected outcomes on day 99, study of early iron supplementation in breastfed infants, Gambia, 2021–2022

CI: confidence interval; GSD: geometric standard deviation.

Notes: For outcomes with log-normally distributed residuals, effect sizes were computed as the ratio of geometric means. A reduction in the soluble transferrin receptor concentration indicates improved iron status.

The haemoglobin concentration was higher in the iron supplementation group than the placebo group at day 99 (crude difference in means: 0.6 g/dL; 95% CI: 0.2 to 1.0), as were levels of other haematological markers, including mean corpuscular volume, mean corpuscular haemoglobin, mean corpuscular haemoglobin concentration, reticulocyte haemoglobin and reticulocyte count (Table 2). There was also a strong indication that the prevalence of anaemia was lower (crude difference: −24.2%; 95% CI: −43.7 to −4.7). Fig. 2 and Fig. 3 illustrate the difference in iron and haematological markers between the groups at day 99. Iron supplementation was associated with an increase of 0.88 SD in the mean reticulocyte haemoglobin concentration and a decrease of 0.70 SD in the mean serum transferrin concentration, compared with a smaller increase in the mean serum iron concentration at 24 hours of 0.53 SD (Fig. 2).There were no marked differences between the groups in the immature reticulocyte fraction, in erythropoietin, α_1_-acid glycoprotein or C-reactive protein concentrations, or in height-for-age, weight-for-age or weight-for-height *z*-scores.

No deaths occurred during the intervention period. Table 3 reports non-serious and serious adverse events, and Table 4 reports events of interest noted by mothers.^14^^,^^15^ There were 10 serious adverse events (five in the iron supplementation group and five in the control group) and 106 non-serious adverse events (54 and 52 in the two groups, respectively), all of which resolved. No event was deemed related to the intervention. All five serious adverse events in the iron supplementation group were infection-related compared with only two in the placebo group. This outcome could have happened by chance. There was no evidence of a difference between the groups in the incidence of maternally reported episodes of diarrhoea, fever, cough, skin infection, eye infection or nasal discharge. However, the incidence of vomiting was lower in the iron supplementation group than the placebo group (difference: 0.37 episodes per 100 child-days; 95% CI: 0.08 to 0.67; Table 4). In line with the low prevalence of *Plasmodium* infection in our study area,^9^ there was no incident case of malaria.

**Table 3:** Adverse events in intention-to-treat population, by intervention, study of early iron supplementation in breastfed infants, Gambia, 2021–2022

Adverse event	Iron supplementation (*n* = 50)			Placebo (*n* = 51)	
	No. events	No. infants affected		No. events	No. infants affected
**Non-serious adverse events**					
Conjunctivitis	1	1		4	4
Fever due to immunization	18	16		25	17
Multiple abscesses	1	1		0	0
Skin rash	6	6		5	5
Upper respiratory tract infection	27	24		16	15
Hospitalization for somatic illness	1	1		2	2
**Serious adverse events**					
Bronchiolitis	1	1		1	1
Constipation	0	0		1	1
Lower respiratory tract infection	1	1		0	0
Pneumonia	1	1		0	0
Sepsis	1	1		0	0
Severe malnutrition	0	0		2	2
Upper respiratory tract infection	1	1		1	1
**Adverse event grade^a^**					
Grade 1	44	29		47	31
Grade 2	7	7		3	3
Grade 3	3	3		2	2

^a^ For grade-1 events, there was awareness of a sign or symptom, which was easily tolerated; for grade-2 events, there was discomfort that was enough to interfere with usual activities; and grade-3 events were incapacitating and led to the inability to perform usual activities.

**Table 4:** Events of interest reported by mothers, by intervention, study of early iron supplementation in breastfed infants, Gambia, 2021–2022

Event of interest and study group	First episode of event			Recurrent new episodes^b^		
	Infants affected, no (%)^a^	Difference between groups, percentage points (95% CI)		Observed incidence^c^	Predicted incidence^b^	Difference in predicted incidence between groups^d^
				No. per 100 child–days (events/child–days)	No. per 100 child–days (95% CI)	No. per 100 child–days (95% CI)
**Diarrhoea**						
Iron supplementation	27 (54.0)	18.7 (−0.3 to 37.8)		1.21 (51/4227)	1.26 (0.81 to 1.70)	0.20 (−0.49 to 0.88)
Placebo	18 (35.3)	Reference		0.93 (39/4194)	1.06 (0.54 to 1.58)	Reference
**Fever**						
Iron supplementation	45 (90.0)	1.8 (−10.4 to 13.9)		2.80 (116/4146)	2.80 (2.24 to 3.35)	0.16 (−0.64 to 0.95)
Placebo	45 (88.2)	Reference		2.64 (108/4088)	2.64 (2.07 to 3.22)	Reference
**Cough**						
Iron supplementation	40 (80.0)	−4.3 (−19.2 to 10.6)		2.94 (120/4083)	3.03 (2.31 to 3.75)	0.37 (−0.60 to 1.33)
Placebo	43 (84.3)	Reference		2.65 (108/4083)	2.65 (2.01 to 3.31)	Reference
**Vomiting**						
Iron supplementation	16 (32.0)	−22.9 (−41.7 to −4.1)		0.43 (19/4470)	0.43 (0.24 to 0.61)	−0.37 (−0.67 to −0.08)
Placebo	28 (54.9)	Reference		0.80 (35/4363)	0.80 (0.57 to 1.03)	Reference
**Skin infection **						
Iron supplementation	17 (34.0)	10.5 (−7.1 to 28.0)		0.65 (29/4430)	0.67 (0.30 to 1.03)	0.32 (−0.08 to 0.73)
Placebo	12 (23.5)	Reference		0.34 (15/4425)	0.34 (0.16 to 0.52)	Reference
**Eye infection**						
Iron supplementation	9 (18.0)	−5.5 (−21.3 to 10.2)		0.27 (12/4484)	0.27 (0.09 to 0.45)	−0.00 (−0.02 to 0.02)
Placebo	12 (23.5)	Reference		0.27 (12/4438)	0.27 (0.14 to 0.40)	Reference
**Nasal discharge**						
Iron supplementation	36 (72.0)	−2.5 (−19.7 to 14.8)		1.94 (82/4233)	1.96 (1.45 to 2.47)	−0.15 (−0.89 to 0.59)
Placebo	38 (74.5)	Reference		2.10 (88/4194)	2.11 (1.57 to 2.65)	Reference

CI: confidence interval; NA: not applicable.

^a^ The sample size for the iron supplementation group was 50 children and for the placebo group 50 children.

^b^ A recurrent new episode was defined, for diarrhoea, as an episode lasting one or more successive days with three or more liquid or fluid stools (i.e. type 4, 5A or 5B), as reported by the mother, separated by at least five successive days without diarrhoea (i.e. type ≤ 3);^16^ for other symptoms, a recurrent new episode was an episode lasting one or more successive days with that symptom, as reported by the mother, separated by at least three successive days without the symptom.

^c^ Calculated as marginal effects from count models with zero inflation, as appropriate.

^d^ All count models used Poisson regression except for skin infection, which was modelled using negative binomial regression; for diarrhoea, cough and nasal discharge, we accounted for excess zero values using zero-inflated regression models.

## Discussion

Our proof-of-principle trial of early iron supplementation in fully breastfed Gambian infants resulted in a substantial improvement in a range of markers of iron and haematological status. Reassuringly, we detected no adverse effects on infections or growth, though a full safety assessment would require a much larger trial. Our study population was typical of many rural African settings where moderate iron deficiency is common among pregnant mothers, and many infants are born small or prematurely, all of which reduce an infant’s iron endowment at birth.

Although iron supplementation soon after birth in infants at risk of iron deficiency is recommended in high-income countries,^6^ in low-income settings supplementation is viewed as potentially undermining the message that exclusive breastfeeding is best.^17^ However, WHO guidance does suggest oral iron supplementation for preterm and low-birth weight infants who are breastfed and have no other source of iron.^18^

Most previous trials investigating the outcomes of early iron supplementation started supplementation at 3 to 4 months of age and assessed haemoglobin or ferritin or both.^19^ Other trials recruited children aged between 1 and 36 months, but did not analyse outcomes separately for the youngest children.^20^^,^^21^ However, one trial in north-east India found that, compared with no iron supplementation, starting supplementation (2 mg/kg as ferrous ascorbate) 36 hours after birth was associated with higher mean haemoglobin (97.0 versus 103.7 g/L, respectively; *P* < 0.0001) and ferritin (78 versus 134 microgram/L, respectively; *P* < 0.001) levels at 6 months.^22^ In China, a four-arm trial of 1276 participants in which iron supplementation was offered in pregnancy or infancy, or both, found that supplementation reduced zinc protoporphyrin levels at 9 months but did not improve other outcomes.^23^ In addition, a trial of infants in Honduras who had been exclusively breastfed for 4 months found that, compared with exclusive breastfeeding to 6 months, the receipt of iron-fortified foods in addition to breastmilk from 4 to 6 months was associated with increased haemoglobin and ferritin concentrations and haematocrit at 6 months.^24^ A similar randomized trial in Iceland found that giving complementary foods in addition to breastfeeding increased serum ferritin concentrations but did not improve levels of haemoglobin or other iron markers.^25^

Given that we were unable to measure the serum iron concentration in young infants over a 24-hour period, the improvement we observed 24 hours after the last iron dose was particularly impressive. A bolus of ferrous sulfate is rapidly absorbed, and serum iron levels in adults peak 3 to 4 hours after administration and decline to baseline after 8 hours.^26^ Hence, in the current study, the iron supply to iron-demanding tissues was probably much larger (an estimated 3- to 4-fold higher) than indicated by the iron concentration measured 24 hours after supplementation. This result is confirmed by the effect of supplementation on the longer-term metrics of iron status. 

The markers of iron and haematological status we used were highly effective in discriminating between intervention and placebo groups at the end of the study. Our findings support the continued use of haemoglobin and serum ferritin measurements, adjusted for C-reactive protein and α_1_-acid glycoprotein levels, in large field surveys,^11^ while noting that the recommended cut-offs are currently under review by WHO. If resources are available for their measurement, mean corpuscular volume and serum transferrin and soluble transferrin receptor levels are effective markers of chronic iron status, and unsaturated iron binding capacity is an effective marker of acute iron status. The reticulocyte count requires specialized haematology analysers and could give misleading results because reticulocytosis is a transient response to improved iron status.

A trial in Honduras and Sweden reported that iron supplementation in breastfed infants was associated with impaired growth.^27^ We failed to find evidence of any such effect and no other trial has replicated this finding.

One strength of our study is that fieldworkers who supervised iron and placebo consumption made daily visits, which ensured high compliance. In addition, fieldworkers asked mothers if their infant was sick, and referred any with worrying conditions to study nurses for adverse event assessment and onwards to a physician for serious adverse events. The main limitation is that this was a proof-of-principle trial with a small sample. A larger sample will be required for replication.

In an ideal world, interventions proven to reduce the risk of a low birth weight and prematurity, to minimize maternal iron deficiency and to improve a baby’s iron endowment (e.g. by delayed cord-clamping) would preclude the need for early iron supplementation of breastfed infants. However, to date these strategies remain poorly implemented in most low-income settings.^28^^–^^30^

A higher haemoglobin level at 6 months of age should enhance an infant’s ability to learn through exploring their environment and should improve attention, although these benefits have been surprisingly hard to demonstrate in studies of older children.^31^ The health and developmental benefits of an enhanced systemic iron supply, as achieved in our study, might be greatest for cognitive and immune function, because critical neural and lymphoid tissues cannot access iron from liver ferritin or haemoglobin (the major body iron stores) and must utilize transferrin-bound iron.

The benefits of early iron supplementation we found are similar to those reported in a meta-analysis of delayed cord-clamping in low- and middle-income countries that assessed ferritin and haemoglobin levels and mean corpuscular volume in children aged between 2 and 12 months.^32^ Although delayed cord-clamping is widely accepted as best practice, it has proven difficult to implement in many settings, particularly, as in our study, where the proportion of home deliveries is high. It would be useful to investigate the combined effect of delayed clamping and early iron supplementation and, possibly, to commence supplementation earlier than 6 weeks.

Following the success of our proof-of-principle trial, further studies are warranted with adequate power to properly assess functional outcomes and safety. Moreover, future studies could help identify those groups of infants who would benefit most from iron supplementation during the early stages of growth and development.
